# Supplementary material for: Leadership Effectiveness in Healthcare Settings: A Systematic Review and Meta-Analysis of Cross-Sectional and Before–After Studies
Source: Int J Environ Res Public Health. 2022 Sep 2;19(17):10995. doi: 10.3390/ijerph191710995 (PMC9518077; doi:10.3390/ijerph191710995)
Supplement: Supplementary file 1 [file ijerph-19-10995-s001.zip › ijerph-1837968-supplementary.pdf]

Supplementary material

All before after studies were conducted in the hospital settings, of which 8 (66·7%) were from public service and 6 (50·0%) were in surgical wards. Trauma care was the most studied specialty (n=3, 25·0%). Multi professional teams (n=8, 66·7%) were involved more frequently than the one professional healthcare workers (n=4, 33·3%). Savage *et al* reported both of outcomes: performance was identified in checklist adherence, surgical quality control procedures and post-operative length of stay; guidelines adherence was in pre-operative treatment adherence and further diagnostic procedure.

First author and type of outcome	Publication year	Continent	Public or private organization	Hospital or Local health Unit	Surgical or medical ward	Multi or single professional	Specialty type	Sample size	Quality score	Year of study conduction
Outcome: guidelines adherence										
Anderson DJ.[38]	2019	North America	private	Hospital	medical and surgical	Multi	Pharmacist and Physicians	13539	12	>2011
Broom J.[34]	2018	Oceania	public	Hospital	surgical	Multi	Surgeon, Nurses, Infectious Dis-Eases/AMS Service, Junior Doctor and Pharmacist	22	10	>2011
Guanche Garcell H.[35]	2019	Asia	private	Hospital	surgical	Single	Surgeon	292	12	>2011
Guzman-Parra J.[36]	2016	Europe	public	Hospital	medical	Multi	Psichiatric	735	11	>2011
Kane M.[32]	2016	North America	public	Hospital	medical and surgical	Multi	Multiprofessional	68	10	>2011
Lewiss RE.[33]	2016	North America	public	Hospital	surgical	Multi	Emergency healthcare workers	1131	7	<2011
Outcome: performance										
Clements A.[29]	2015	Oceania	public	Hospital	surgical	Single	Emergency nursing staff	40	4	>2011
Davies C.[40]	2019	Europe	private	Hospital	medical and surgical	Single	Nurse	1254	8	>2011
Murphy M.[37]	2018	Oceania	public	Hospital	surgical	Multi	Trauma team (Doctors, Nurses, Allied Health)	1273	11	<2011
Stargell LF[41]	2018	North America	private	Hospital	medical	Multi	Senior Leader	3600	8	>2011
Weech-Maldonado R.[39]	2019	North America	public	Hospital	medical and surgical	Multi	-	287	5	<2011
Outcome: guidelines adherence and performance										
Savage C.[31]	2017	Europe	public	Hospital	surgical	Single	Pediatric Surgeon		11	<2011

Table S1. Before after studies included in this systematic review and meta-analysis.

The forest plot in Supplementary Figure 1 represented the interventions effectiveness of each four leadership styles on healthcare outcomes. Transformational leadership style showed the highest outcome improvement with an increase of 27% (95%CI 12-42%), followed by transformational leadership style (24%; 95%CI 17-30%) and servant leadership style (13%; 95%CI 8-17%).

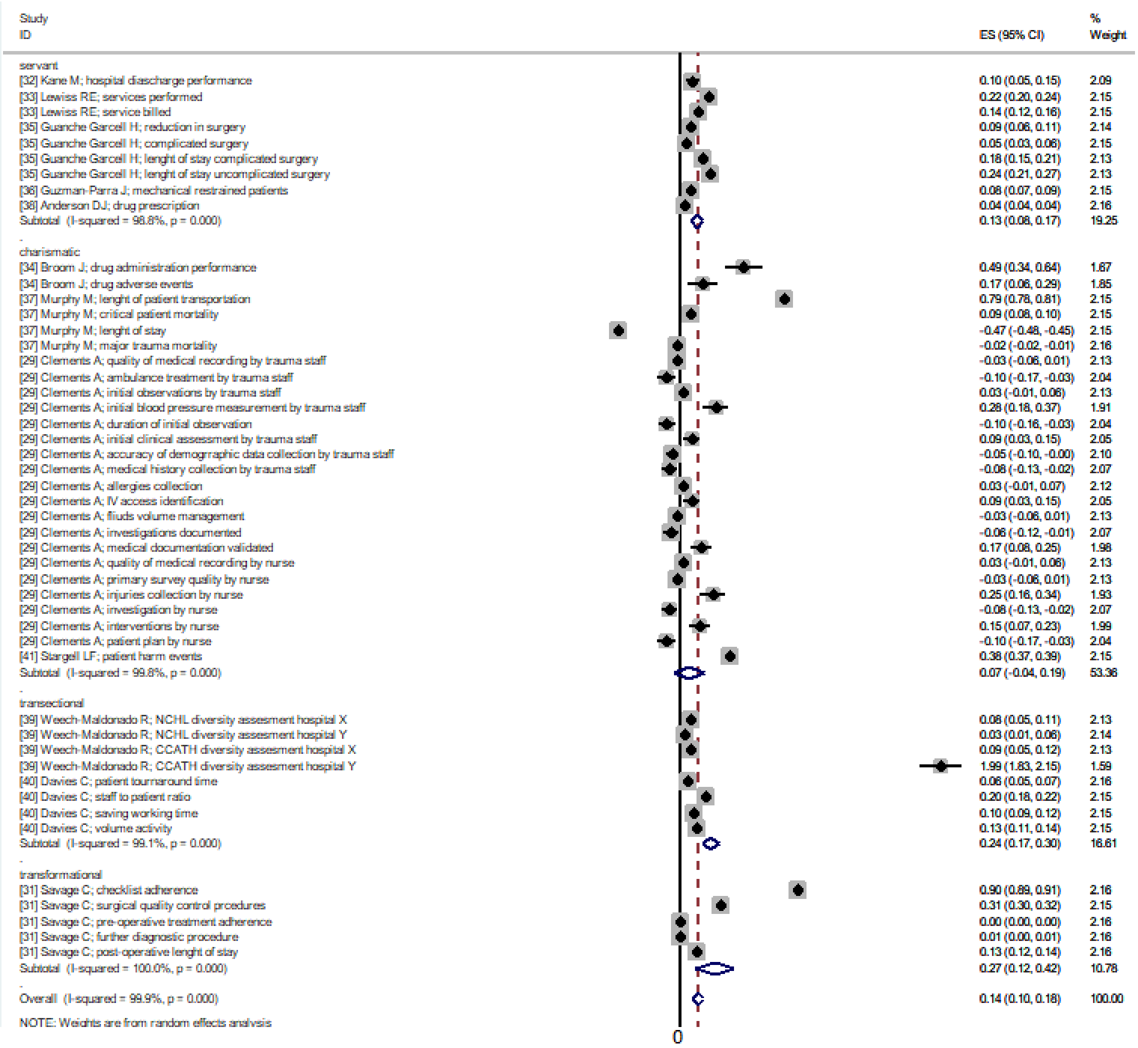

Figure S1. *Leadership effectiveness by leadership style in before after studies.*

Funnel plot for publication bias showed asymmetry among before after studies (Supplementary Figure 2).

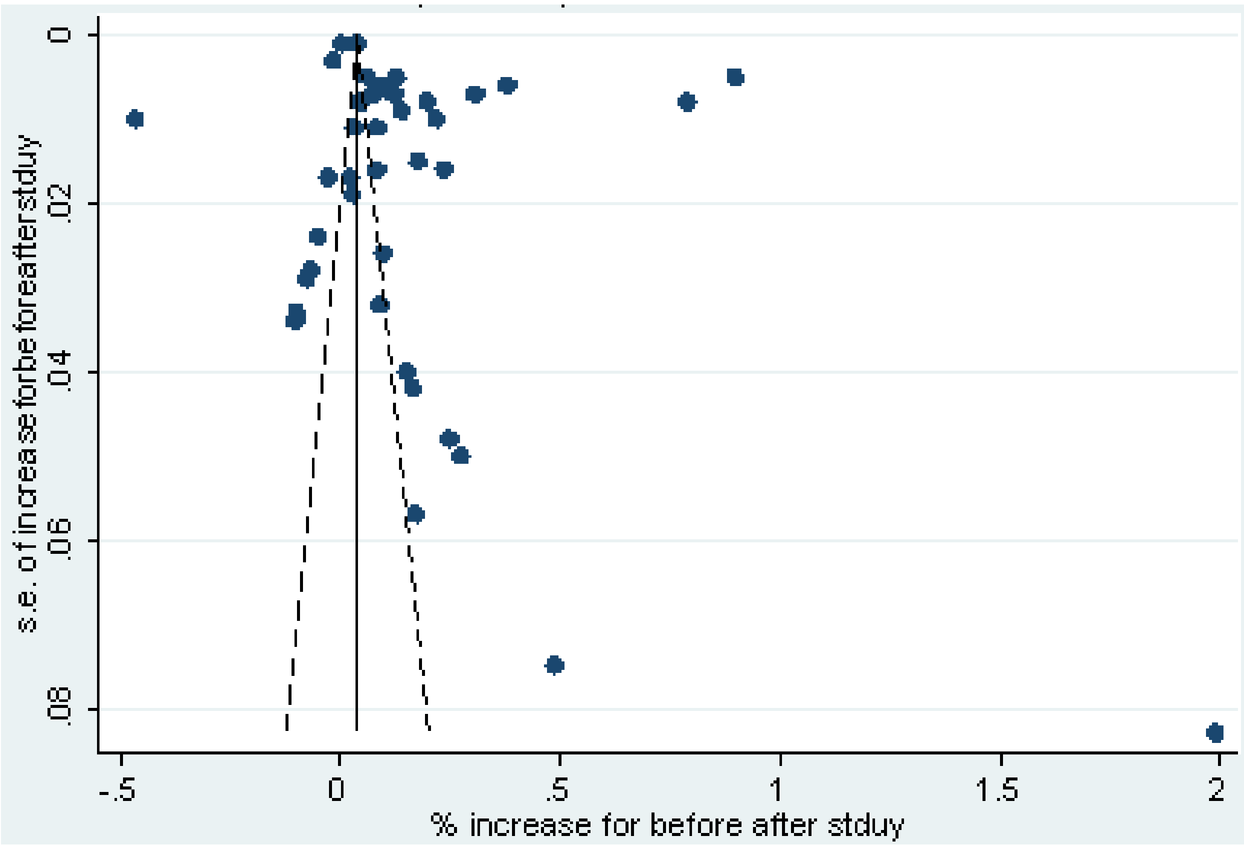

Figure S2. *Funnel plot of before after studies.*

Most of cross-sectional studies (Supplementary table 2) were conducted in hospital setting (n=7, 77·8%), while local health units (LHU) were analyzed in 2 articles (22·2%). Almost all studies were conducted within public healthcare organizations (n=8, 88·9%).

First author	Publication year	Continent	Public or private organization	Hospital or local health unit	Surgical or medical ward	Multi or single professional	Specialty type	Sample size	Quality score	Year of study conduction
Asiri SA;[49]	2016	Asia	Public	hospital	medical and surgical	single	Acute care unit	332	8	>2011
Barling J;[47]	2018	North America	Public	hospital	surgical	multi	Surgery, Urology, Obstetrics and Gynecology	150	9	>2011
Gong Z;[43]	2019	Asia	Public	hospital	medical and surgical	multi	Nursing care	458	10	>2011
Hageman MGJS;[42]	2015	North America	Private	hospital	surgical	single	Orthopaedic surgeons	26	4	>2011
Kara A;[46]	2015	North America	Public	hospital	medical and surgical	multi	Nurses, Case managers, Pharmacists, Nutritionists, Medical students, Social workers	110	7	>2011
Kim H;[45]	2019	Asia	Public	hospital	medical and surgical	multi	Nursing care	199	8	>2011
Kim MH;[44]	2019	Asia	Public	hospital	medical and surgical	multi	Nursing care	324	10	>2011
Lornudd C;[30]	2015	Europe	Public	local health unit	medical and surgical	multi	Not specified healthcare workers	1249	10	<2011
Marin GH;[48]	2015	SouthAmerica	Public	local health unit	medical	multi	Primary care	10	9	<2011

Table S2. *Cross-sectional studies included in this systematic review and meta-analysis. All outcomes were performance.*

Leadership style analysis reported in the forest plot (Supplementary Figure 3) showed that transectional leadership had the highest increase (0·48; 95%CI 0·14-0·82), followed by servant leadership style (0·20; 95%CI 0·14, 0·25). In detail, the study with the greatest increase was Marin GH *et al*, whose outcomes were good purchased with increase of 0·97 (95%CI 0·85-1·09) and invoiced with 0·95 (95% CI 0·79, 1·10). Member-leader turnover intention by charismatic leadership style was the worst outcome, decreasing leadership effectiveness with a correlation coefficient of 0·46 (95% CI 0·54- -0·38).·

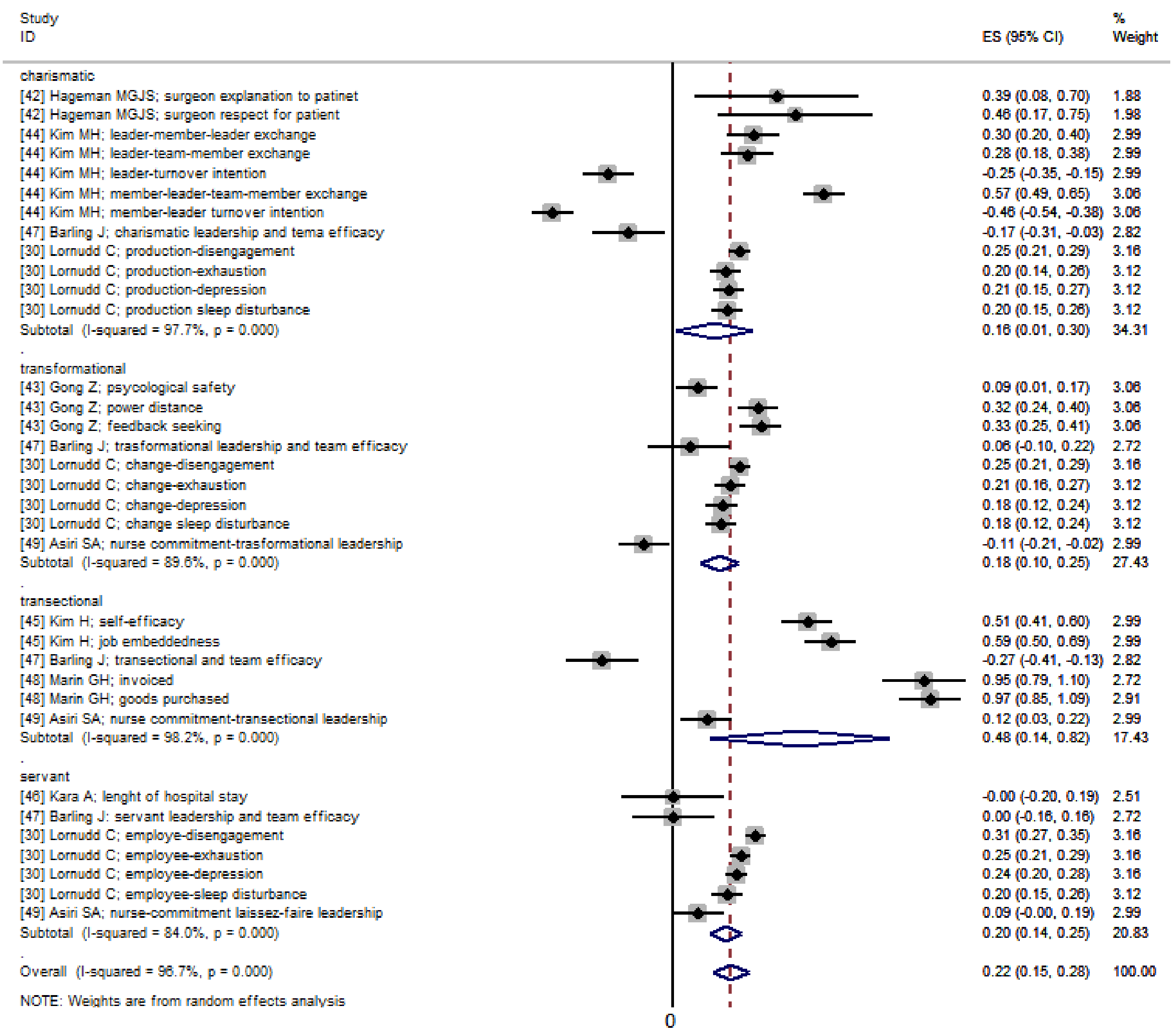

Figure S3. *Leadership effectiveness in cross sectional studies by four leadership style.*

Asymmetry and some outliers were checked in the funnel plot for cross-sectional studies (Supplementary Figure 5).

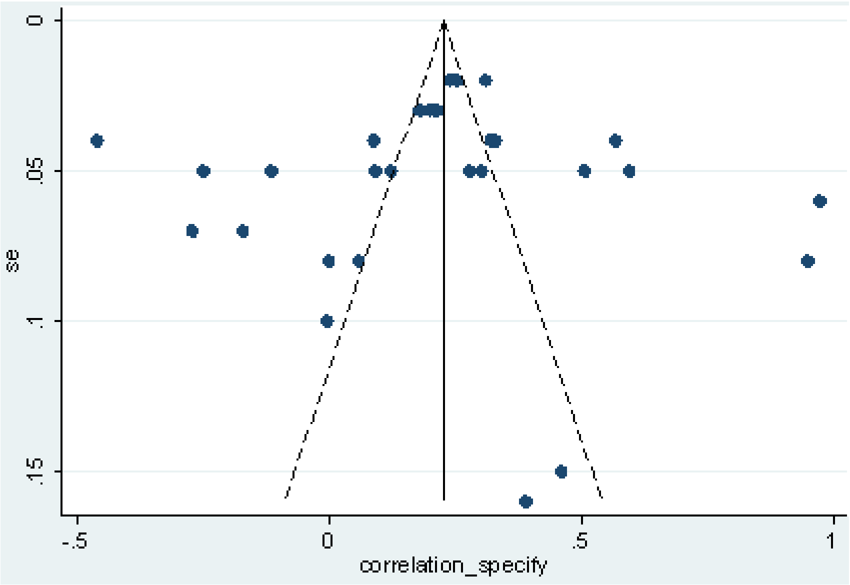

Figure S4 *Funnel plot of cross-sectional studies.*
